# Temperature Based Process Characterization of Pharmaceutical Freeze-Thaw Operations

**DOI:** 10.3389/fbioe.2021.617770

**Published:** 2021-04-09

**Authors:** Dennis Weber, Jürgen Hubbuch

**Affiliations:** Institute of Engineering in Life Sciences, Section IV: Biomolecular Separation Engineering, Karlsruhe Institute of Technology (KIT), Karlsruhe, Germany

**Keywords:** freeze-thaw, process characterization, freezing time, formulation, last point to freeze

## Abstract

In biopharmaceutical production processes, freeze-thaw operations are used to ensure product integrity during long hold times, but they also introduce additional stresses such as freeze concentration gradients that might lead to a loss of protein activity. Process characterization of freeze-thaw operations at different scales should be conducted with attention to freezing time and boundary effects to ensure the product stability throughout the process and process development. Currently, process characterization often relies on one or very few temperature probes that detect freezing times based on raw temperature, which is largely influenced by freezing-point depression in case of concentrated solutions. A method to detect freezing based on the second derivative of temperature measurements from Fiber-Bragg-Grating sensors is presented to overcome this issue. The applicability of the method is demonstrated by process characterization of a novel small-scale freeze-thaw device with minimized boundary effects using freezing times of purified water and concentrated formulations. Freezing times varied from 35 to 81 min for temperatures between −60 and −20°C and impacted freeze concentration profiles. Furthermore, freezing time estimations based on the Plank equation revealed model limitations due to start-up temperature gradients, that can be corrected by an empirically extended Plank model. As a hypothesis, we conclude that freezing temperature, from a freeze concentration view, is less important in containers with small characteristic freezing distances such as freeze bags. Using a 2D-resolved temperature profile, a shift of the last point to freeze position from top to bottom of a container was observed when freezing above −30°C.

## Introduction

Therapeutic proteins are among the top selling pharmaceuticals. Due to their high value and production cost, activity loss of the active pharmaceutical ingredient (API) during shipment and storage has to be limited by selection of suitable formulation agents (Chang et al., 2005; Bauer et al., 2017) and storage conditions. Therefore, many biopharmaceuticals are stored in a frozen state (Singh et al., 2009; Authelin et al., 2020). While freezing slows down and reduces degradation reactions of the API, freezing processes expose the protein to different stresses such as cold denaturation (Privalov, 1990), freeze concentration (Bhatnagar et al., 2007), ice crystal formation (Chang et al., 1996), and potential excipient crystallization. Protein activity loss or aggregation was correlated with freeze concentration (Reinsch et al., 2015; Roessl et al., 2015). At a microscopic scale, crystallization of water molecules leads to freeze concentration of the remaining solutes within the ice crystal structure causing freezing temperature dependent phase behavior of proteins (Wöll et al.,2019a,b). In large scale freezing operations, macroscopic freeze concentration leads to non-homogeneous solute distribution profiles. During freezing of larger bulk volumes, ice fronts progress from cooled container walls toward the center of the container. While freezing, solutes are concentrated in front of the phase boundaries and partition between solid and liquid phase (Bhatnagar et al., 2007), leading to macroscopic freeze concentration. Additionally, this freeze concentration effect leads to natural convection due to density gradients (Butler, 2002) and therefore settlement of solutes. As a result, with the occurrence of freezing fronts, a typical freeze concentration profile with the peak concentration at the center bottom of a frozen bulk is unavoidable (Maity et al., 2009; Kolhe and Badkar, 2011; Roessl et al., 2014; Reinsch et al., 2015).

Freeze concentration was shown to be dependent on the freezing process rather than storage temperature (Hauptmann et al., 2019). However, investigations of freeze-thaw characterizations with a focus on heat transfer and phase change are missing (Fan et al., 2018), but necessary to ensure process scalability and to allow process optimization with regards to ideal freezing temperature. A key parameter often used for characterization of freezing processes is the ice front velocity, which impacts the maximum freeze concentration (Webb et al., 2002; Rodrigues et al., 2011; Hauptmann et al., 2019) and the total freezing time. The freezing time determination is commonly based on crude temperature measurement at the last point to freeze (LPTF), where the ice fronts come together at the end of a freezing process. This calculation relies on the assumption of ice below the freezing point temperature which is often arbitrarily set from −0.5 (Rodrigues et al., 2011) to −5°C (Lashmar et al., 2007) due to freezing point depression occurring in highly concentrated solutions. Furthermore, the temperature probe has to be exactly at the LPTF position. A method for determination of the freezing time independent from freeze point depression and the LPTF position has not been presented. In a recent publication, the Plank equation was suggested to model total freezing times of pharmaceutical processes (Authelin et al., 2020), which allows for process time prediction and optimization. To date, a validation of the model in pharmaceutical freezing processes, however, is missing (Authelin et al., 2020).

In the following work, a derivative-based temperature analysis for the detection of the total freezing time is presented. This method is used for process characterization of a novel small-scale freeze-thaw model with regards to freezing times at different temperatures. The applicability of the Plank equation on actively cooled pharmaceutical freezing processes is discussed to improve transferability of freezing processes to different scales and freezing setups. An extension of the Plank equation is introduced for correction of transient start-up conditions.

## Materials and Methods

### Sample Preparation

All used solutions were prepared with ultrapure water (PURELAB Ultra, ELGA LabWater, Veolia Water Technologies, Saint-Maurice, France) and sterile filtered using a 0.2 μm filter prior to application. As Tris-(hydroxymethyl)-aminomethane (TRIS) buffer is a widely used buffer in protein formulations (Zbacnik et al., 2017), TRIS buffer was prepared from TRIS purchased from Merck (Darmstadt, Germany) and Tris-(hydroxymethyl)- aminomethane-hydrochloride purchased from AppliChem (Darmstadt, Germany) at a concentration of 500 mM. The pH was adjusted to pH 7.5 ± 0.1 using hydrochloric acid. A solution with a single component was selected to reduce component interactions and elevated concentrations were chosen for better comparability with highly concentrated pharmaceutical formulations that are usually frozen for shipment. Furthermore, freezing point depression is dependent on solute concentration, which emphasizes the need for new method for total freezing time calculation. Similar concentration effects are expected for varying solutes such as proteins and stabilizing sugars (Rodrigues et al., 2011).

### Novel Small-Scale Freeze-Thaw Device

**FIGURE 1:**
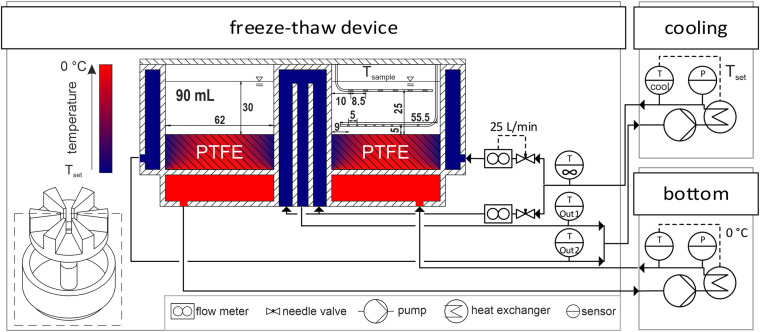
Experimental setup. A piping and instrumentation diagram of the cryogenic device with the two individual cooling units to control the freezing chambers is displayed. Freezing chambers are visualized as a cross-section of the small-scale freeze-thaw device. Temperatures of cooling fluids and PTFE inlay are indicated by a gradient from T_*set*_ to 0°C in blue to red, respectively. Inside the cross-section, sample temperature probes and dimensions in mm are shown as gray spots on the bent steel encapsulations in white. Pipes are represented by arrows. Hatched areas indicate solid stainless steel and PTFE.

A novel small-scale freeze-thaw device was designed and manufactured together with Bilfinger Industrietechnik Salzburg GmbH, Schwetzingen, Germany. It is designed as a scale down model of an industrial scale freezing unit representing a slice of a larger hollow tube. The hollow cylindrical shaped freezing unit, depicted in Figure 1, is cooled by an outer cooling jacket and an inner cooling tubing, while the bottom is heated separately to minimize scale-down boundary effects. The container volume is divided into six individual chambers by an inlay made from polytetrafluoroethylene (PTFE). This inlay allows to perform up to six individual experiment at a working volume of up to 90 mL per chamber. Furthermore, the PTFE also balances the heat fluxes at the bottom of the chamber due to the low heat conductivity resulting in a temperature profile as illustrated in cross-section in Figure 1. This PTFE bottom counteracts boundary effects present due to heat conductivity of the steel walls. The outer and inner cooling walls are made from 3 mm thick, 316L stainless steel and have a radius of 38 and 100 mm, respectively. The chamber depth is 40 mm. All tubing and steel parts are insulated using 20 mm Armaflex from Armacell (Münster, Germany).

A piping and instrumentation diagram of the freeze-thaw device is depicted in Figure 1. In order to achieve the desired temperatures, the inner and outer cooling circuits of the device were merged and connected to the cryogenic device Integral XT 1590 with the cooling fluid Kryo 90, both purchased from Lauda (Lauda-Königshofen, Germany). With installed flow meters from Krohne (Duisburg, Germany), needle valves and a set system pressure of 2 bar, a cooling fluid flux of 25 ± 5 L/min was achieved in the inner and outer circuit. The bottom of the container was heated by the cooling unit F25-MC used with the cooling fluid Thermal HY both purchased from Julabo (Seelbach, Germany). In a preliminary study, a constant bottom temperature of 0°C was found to be ideal for minimal boundary effects indicated by parallel freezing fronts in the container, when freezing double deionized water (data not shown). All units where operated at maximum power without any set temperature gradients. PT100 thermoelements connected to a datalogger ALMEMO 8590 by Ahlborn (Holzkirchen, Germany) measured the cooling fluid temperatures on-line at the inlet of the freeze-thaw device before stream division and at the two outlet streams from the inner and outer circuit. The setup allowed for set temperatures from −60 to 30°C. Measured data were collected and controlled using a custom app designed with MATLAB App Designer by Mathworks (Natick, MA, United States). The app automatically collected and set all temperature data on-line from the datalogger, the two cooling units and the sample temperature device described above. In the following sections, the cooling temperature T_*cool*__*ing*_ refers to the temperature of the Kryo 90 cooling unit, while the bottom temperature was set to a constant temperature of 0°C for all experiments. The fluid temperature T_∞_ refers to the measured temperature at the inlet of the cryogenic device. The set temperature T_*set*_ is the temperature achieved in the cooling unit (T_*cooling*_) after cooldown.

Prior to an experiment, the gaps between the PTFE inlay and the housing were sealed using food grade silicone Ottoseal S27 from Otto-chemie (Fridolfing, Germany). For each experiment, 90 mL of the sample was pipetted into a chamber. Neighboring chambers were filled with ultra-pure water to minimize radial boundary effects. A freezing process started with a hold phase at 5°C for at least 2 h to assure equilibrium starting conditions. After equilibration, the set temperature was adjusted between −60 and −20°C at maximum cooling ramp. After all sample temperature sensors measured temperatures below −1°C, the bottom heating unit was turned off automatically to achieve minimal sample temperatures. If frozen samples were taken, a hold time of 2 h was added post-freezing to assure that the freezing process was at equilibrium. After the freezing step, thawing was initiated by setting the temperature to 30°C for at least 1 h. Timing of the different phases was automated using the app described above.

### Temperature Evaluation

The sample temperature was monitored using 14 temperature sensors as shown in Figure 1 with information on the probes’ coordinates. The sensors were located on two pre-calibrated optical temperature fibers in a custom design from Loptek (Berlin, Germany) together with the interrogator SCN-46 S-line Scan 416 from Sylex (Bratislava, Slovakia). These fibers were encapsulated by a stainless-steel tubing of 1 mm diameter and contained up to nine Fiber-Bragg-Grating sensors. One fiber with six sensors was positioned 5 mm below the sample surface and a second fiber with eight sensors was positioned 5 mm above the ground of the chamber to achieve a 2D-resolved temperature field of a chamber cross-section. Temperature data were obtained every 2 s using S-line Sentinel Software from Sylex (Bratislava, Slovakia). The used temperature monitoring set-up provides the benefits of increased temperature resolution while reducing heat conduction through the sensor cables when compared to commonly used thermoelements. We do not expect, that heat conduction along the sensors influences the freezing process, as the heat capacity of the hollow 1 mm thick tubes is negligible compared to the surrounding ice and water.

**FIGURE 2:**
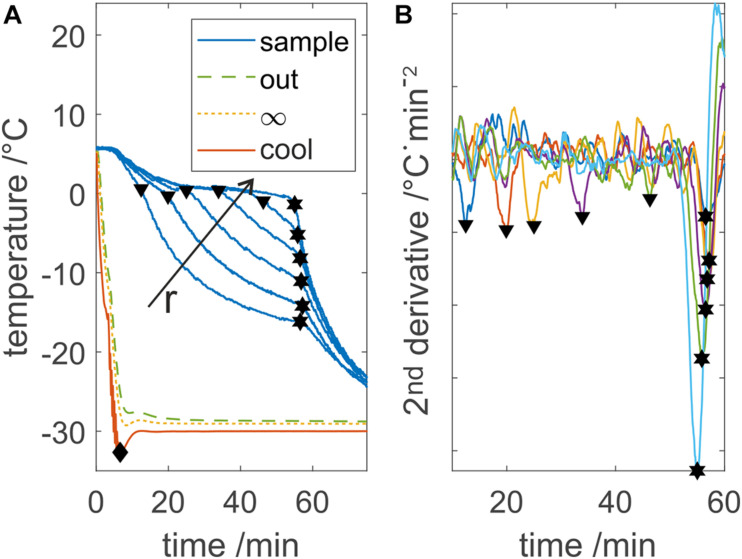
Process temperatures evaluation example. **(A)** Displays the measured device temperatures T_*out*_, T_∞_, and T_*cool*_ and five out of 13 sample temperatures. The diamond shape indicates the cooldown time, triangle shows the freezing at an individual probe, and hexagons show the LPTF. Freezing at individual probes and the LPTF time are calculated based on minima in the second derivative of the sample data, which is shown in panel **(B)**. r indicates the increasing distance of the temperature probe from the cooling surface. Individual sample temperature profiles are colorized in panel **(B)** for visibility.

Looking at a typical sample temperature profile as shown in Figure 2A, two distinct phase transition times can be detected. The first transition occurs upon freezing at the observed temperature probe, where the temperature drops below the melting point, referred to as partial freezing time. At this point, thermodynamic properties change from water to ice, leading to a distinct variation of the temperature slope. The second event indicates freezing at the LPTF, which is referred to as total freezing time. When the entire bulk is frozen, no more latent heat will be released and thus, temperature at all probes will decrease simultaneously. For analysis of the two times measured with each temperature probe, the temperature profiles were smoothed and derived twice using a Savitzky–Golay-Filter, with a second order polynomial and a window of 151 data points. Minima in the second derivative indicate the discussed slope changes. The filter window should be carefully selected based on the data quality as discussed later, where smaller windows result in higher precision but also higher signal to noise ratio. The freezing time was calculated based on all 14 temperature measurements, with outliers based on three scaled median absolute deviations removed to improve calculation robustness. However, partial freezing times were determined based on temperature below −2°C, due to the low signal to noise ratio of the used fiber optic sensors. When this method was applied to temperature readings from thermoelements, both partial and total freezing time could be analyzed based on second derivative with a smaller Savitzky–Golay-Filter window.

The cooldown time until the cooling fluid reached the set temperature was calculated based on the minimum temperature of the set temperature.

### Freezing Time Prediction

Plank’s model to calculate the total freezing time t_*freezing*_ (Plank, 1913), was suggested by Authelin et al. (2020) as a model in pharmaceutical freezing processes. It assumes a step decrease of the cooling fluid temperature T_*cooling*_ from equilibrium to set temperature and the bulk to be at melting temperature T_*m*_ prior to freezing. The equation can be written as

(1) $$t_{P\,l\,a\,n\,k}=\frac{\rho_{l}\,\Delta \,h_{m}\,\,X}{E\,\,k_{a}\,\,\left(T_{m}-T_{c\,o\,o\,l\,i\,n\,g}\right)}\,\,\left(1+\frac{B\,i}{2}\right)$$

where ρ_*l*_ is the density of the liquid sample, Δh_*m*_ is the latent heat of fusion and *X* is the characteristic distance between the LPTF and the heat transfer surface (Pham, 2005). The geometric factor E is 1 for infinite slabs, 2 for infinite cylinders and 3 for spheres. The Biot number Bi = k_*a*_X/λ_*s*_ relates the heat transfer resistance of the shell k_*a*_ to internal heat conductivity resistance of the sample with a given heat conductivity λ_*s*_ (Martin, 2010). For pharmaceutical solutions, the physical properties of solid water as the solvent can be used under the assumption of diluted solutions (Randall and Rossini, 1929; Kumano et al., 2007). Assuming a negligible temperature difference between the equilibrium and melting temperature and the wall temperature equals T_∞_ (Bi →∞), which may be done for actively cooled freezing systems, the freezing time may be calculated using Eq. 2

(2) $$t_{f\,r\,e\,e\,z\,i\,n\,g}=\frac{\rho_{l}\,\Delta \,h_{m}\,\,X^{2}}{2\,\,E\,\,\lambda_{s}\,\,\left(T_{m}-T_{c\,o\,o\,l\,i\,n\,g}\right)}=\frac{\beta }{-T_{c\,o\,o\,l\,i\,n\,g}}\,$$

where β in °C⋅min is a function of ρ_*l*_, Δh_*m*_, X^2^, λ_*s*_, and E, summarizing all constant values for a given active freezing system.

### Frozen Bulk Analytic

A hollow drill from Bürkle (Bad Bellingen, Germany) with an inner diameter of 8 mm has been used to take samples from the frozen bulk. A 3D-printed mount with nine drill holes in two different rows at an angle of 10.5° was placed on top of a chamber, providing reproducible drill positions across the whole chamber length. Under the assumption of negligible radial boundary effects, sampling from two different rows with overlapping sample volumes increased the sample resolution of a cross section as depicted in Figure 1 in solid and dashed lines. Samples were taken from the drill holes at three levels of 8 mm height with the last hole 2 mm above the chamber bottom. The sample above the first sample level has been discarded at all times to avoid uptake of ice fragments from the previous drilling and measuring of the freeze concentrated liquid from the center of the chamber that was pushed out where the expanding ice fronts met. The samples were transferred into 2 mL reaction tubes from Eppendorf (Hamburg, Germany) and thawed at room temperature. The buffer concentration of the samples was measured by analysis of the conductivity using a conductivity meter CDM 230 from Radiometer Analytical SAS (Lyon, France).

## Results

### Freezing Time Analysis

**FIGURE 3:**
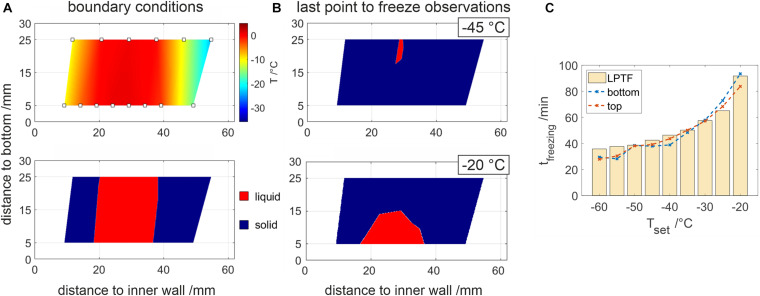
2D resolved temperature analysis of the chamber cross-section. **(A)** displays interpolated temperature data, where temperature probes are indicated by rectangles. In the lower figure, temperatures below –1°C and above are highlighted by blue and red, respectively. Panel **(B)** shows highlighted temperatures prior to the LPTF for –45 and –20°C. **(C)** Comparison of the individual freezing times at the bottom and top temperature sensor at different set temperatures. LPTF times determined by the temperature slope are plotted as bar plots for reference.

In order to characterize and describe a freezing process, the total freezing time is a key process parameter. Total freezing times could be calculated from the second derivative as shown in Figure 2. While minima at the total freezing time are prominent, minima at the partial freezing time are not. With elevated freezing temperatures above −25°C, the first minima were not detected correctly at all temperature probes due to low signal to noise ratios of the temperature sensors. Therefore, the partial freezing times were determined when the temperature fell below −2°C. With the freezing times at the individual probes, ice front progression in the chamber could be monitored as presented in Figure 3. Total freezing times calculated from the second derivative of all temperature profiles resulted in standard deviations below 2 % for pure water samples and below 4 % for highly concentrated buffer samples. Looking at observed process temperatures shown in Figure 2A, the cooling temperature exceeded the set temperature by up to 3 K due to temperature regulation, whereas the fluid temperature did not, due to heat capacity of the steel housing.

### Process Characterization

**FIGURE 4:**
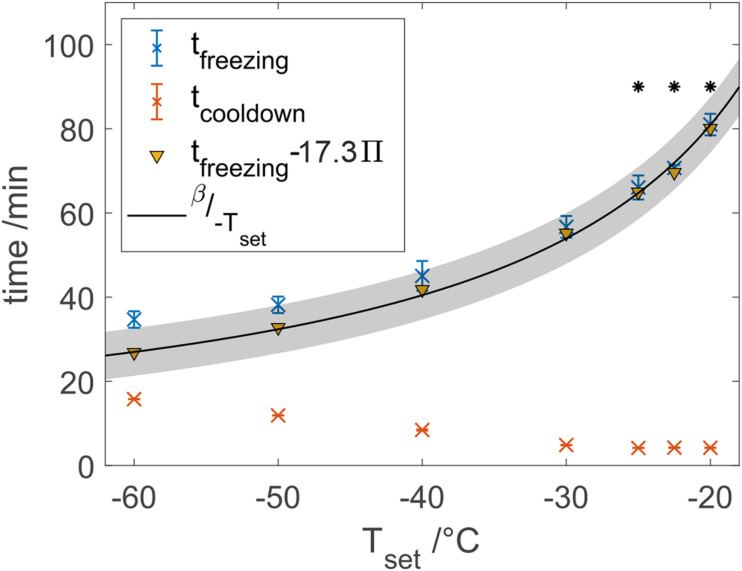
Freezing and cooldown times at different set temperatures. Data marked points with a star were used to calibrate a model after Eq. 2 with β = 1619, where the gray area shows the 95% confidence bounds.

Purified water was frozen at different temperatures to characterize the novel freeze-thaw device with respect to heat transfer properties because of the availability for large scale process characterization. In our studies, the total freezing time of water samples shortened by 2.3-fold from 78.7 to 33.8 min when decreasing the set temperature from −20 to −60°C as shown in Figure 4. Freezing times shortened faster at higher cooling temperatures compared to an almost stagnant freezing time reduction at lower cooling temperatures. Based on Eq. 2, an estimation model has been fitted using data from −25 to −20°C, which resulted in a β-value of 1619°C⋅min with *R*^2^ = 0.972. Solving Eq. 2 with the data at −20°C results in β = 1620°C⋅min. A root-mean-square error (RMSE) of 1.83 min for the model and all set temperatures was calculated, where the measured freezing times at temperatures below −25°C always exceeded the estimated freezing time. A ratio of cooldown time and total freezing time Π was calculated after Eq. 3. Subtraction of empirically determined 17.3-fold Π from the total freezing times reduced the RMSE to 0.96 min, where especially the estimation of freezing times at lower temperatures was improved as indicated in Figure 4.

(3) $$\Pi =\frac{t_{\text{cooldown}}}{t_{\text{freezing}}}$$

Analyzing the temperature of the heat transfer fluid inside the cooling unit, cooldown times of 4.2–4.9 min for set temperatures of −30°C and above were measured. Cooldown times greatly increased for lower set temperatures. The temperature differences between cooling fluid and the cryo-device outlet were up to 3 K after freezing process start and reduced to around 0.5 K at equilibrium state. Using a mean heat capacity of 1.5 kJ/kg/K and a density of 900 kg/m^3^ of the heat transfer fluid provided by the supplier and a flow rate of 25 L/min, a power loss of 330 to 100 W can be estimated at equilibrium state.

### Sample Temperatures

With the help of temperature fibers, a continuous two-dimensional resolved temperature field could be observed as shown in Figure 3. Assuming frozen sample for temperatures below −2°C, the ice front progression was observed during a freezing process. When freezing 500 mM Tris buffer solution, perpendicular freezing fronts have been observed as shown in Figure 3A. The LPTF was observed at a distance of 26.5 ± 2.5 mm from the inner cooling wall at all experiments. In contrast, the vertical position of the LPTF varied with temperature as shown in Figure 3B. In experiments with set temperatures below −25°C, the freezing fronts met at the bottom of the container first and a small gap froze from the bottom to the top of the container, resulting in a LPTF position at the top of the chamber. This occurs as liquid is pushed to the bulk surface by the expansion of water molecules, which agrees with an observed iceberg on top of the sample bulk at the location of the LPTF. Freezing at elevated set temperatures of −25°C and above resulted in a LPTF position at the bottom of the container, where the remaining liquid froze in the form of a shrinking bell. Figure 3C shows total freezing times at the top and the bottom of the container based on temperature compared to the total freezing times calculated from temperature slopes. The slope based freezing times were similar to those observed for purified water shown in Figure 2. Differences in freezing times at the top and bottom of the freezing container were marginal for set temperatures below −25°C when compared to differences of up to 9.7 min at higher set temperatures.

### Frozen Bulk Analysis

**FIGURE 5:**
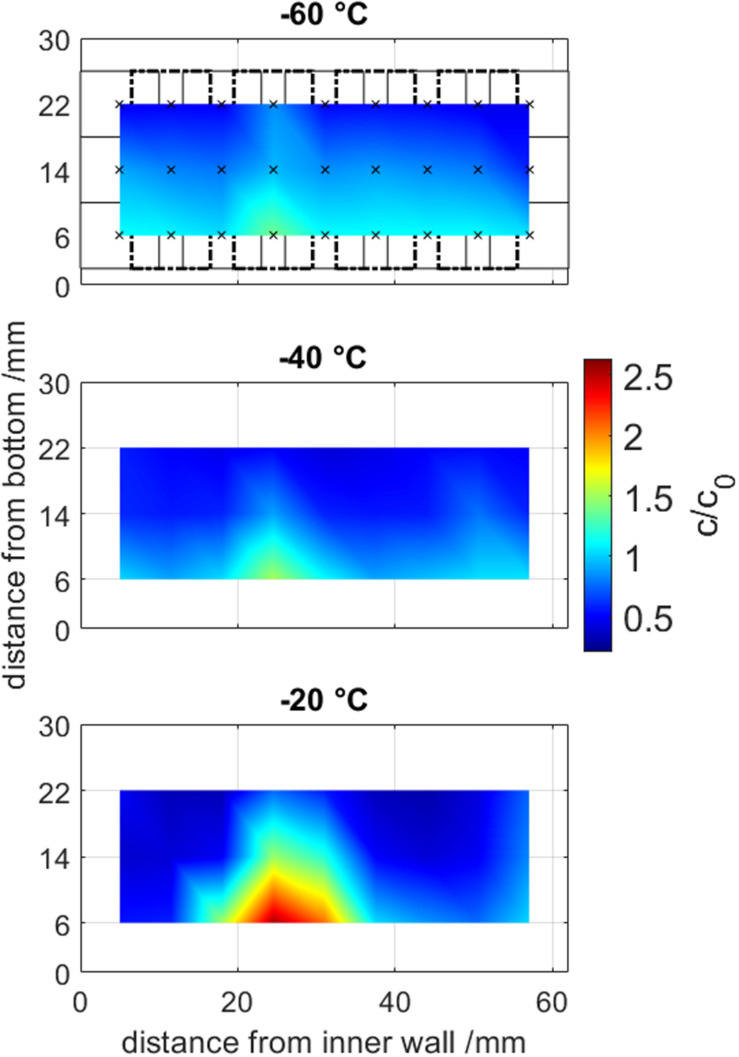
Offline sample analysis from frozen drill cores at temperatures from –60 to –20°C. Samples volumes are visualized in the first graph in solid and dashed rectangles, where the center of a measurement is marked by an x. Normalized concentration are visualized by a color gradient from blue to red and are interpolated between the averages from triplicates (*n* = 3).

A 500 mM Tris buffer solution at pH 7.5 has been frozen at −20, −40, and −60°C to evaluate the influence of the freezing temperature on cryo-concentration and the results are shown in Figure 5.

The maximum freeze concentrated area was located at the LPTF position 25 mm away from the inner cooling wall at all freezing experiments. When increasing the temperature from −60 to −40 to −20°C, the maximum freeze concentration increase (c_*max*_/c_0_) rose from 1.39 ± 0.01 to 1.52 ± 0.03 to 2.53 ± 0.04, respectively. The bulk inhomogeneity, more specifically the ratio of the maximum to the minimal concentration (c_*max*_/c_*min*_), summarizes freeze concentration results and high values represent high freeze concentration. The bulk inhomogeneity improved significantly when lower the freezing temperature from 8.1-fold to 3.6-fold to 3.0-fold at freezing temperatures of −20, −40, and −60°C, respectively. In general, it was observed that a concentration gradient from top to bottom was present. The authors want to mention, that the sampling method only provided averaged concentrations of the sample volume, whereas the true local peak freeze concentration is expected to be higher.

## Discussion

### Freezing Time Analysis

We demonstrated a method to extract freezing times at individual positions of temperature probes and the total freezing time from the second derivative of various temperature profile. A common problem in the detection of freezing at individual probes is freezing-point depression which leads to assumptions such as solid state below −5°C (Lashmar et al., 2007). Furthermore, detection of the total freezing time based on raw temperature profile relies on the knowledge of the LPTF position. We overcome both of these issues with the approach to use the second derivative of the temperature. However, the method relies on precise and high frequency data with high differences in freezing and equilibrium temperature. With higher freezing temperatures, signal to noise ratios decreased due to lower absolute temperature gradients. Thus, standard deviations of freezing times increase with higher freezing temperatures. As a result, only LPTF freezing times were calculated by slope analysis. Individual freezing times used to calculate freezing times at the top and bottom of the container were assumed for temperatures below −2°C.

Comparing the two methods as shown in Figure 3C reveals deviations of the two methods. While derivative-based analysis shows similar trends seen for purified water, temperature-based analysis often leads to larger deviations from expected freezing times. Nonetheless, temperature-based analysis was able to indicate settling of the LPTF to the ground of the chamber as discussed in the following sections. This can be important when designing freezing processes with a single thermoelement, as this should be directed at the LPTF. Unfortunately, the fiber optic temperature sensors provided a generally lower signal to noise ratio when compared with common thermo elements. This, however, shows the applicability and robustness of the derivative based method.

### Extensions of the Plank Equation

As shown above, a decrease of the freezing temperature shortens total freezing times as expected. The non-linear trend can be explained partially by thermodynamic properties of freezing processes as we showed by our model based on Plank’s model. The calculated model based on three initial freezing tests with water was able to estimate total freezing times at lower temperatures.

With lower freezing temperatures, however, larger deviations of our experimental results from the Plank model were observed. These deviations show the process related limitations of the Plank equation. Unlike our model, the measured values are expected to approach a minimal freezing time of approximately 31 min for lower freezing temperatures, based on exponential extrapolation of all measured data. The rising cooldown times for decreasing temperatures could explain the limited behavior of the real freezing time since the Plank equation is only applicable to step-like cooling temperatures.

Therefore, a dimensionless number Π was introduced to take the transient starting conditions of a freezing device into account. For an ideal system, with a step like cooling temperature decrease, Π approaches 0, whereas in real systems, Π is expected to increase with decreasing freezing temperatures due to physical heat transfer restrictions in cooling units. If it exceeds 1, lowering the set cooling temperature will not result in a further decrease of cooldown times as the initial transient period exceeds the freezing time. Hence, cryo-concentration of solutes will not decrease either. Π values with our setup are summarized in Table 1. Π rises with increasing cooldown times at lower set temperatures as more enthalpy needs to be removed from the cooling fluid and as cooling units become less efficient at lower temperatures. Empirical correction of freezing times by the calculated Π values significantly improved the model. We therefore conclude that the Plank equation may be used to predict freezing times even at low temperatures when transient start-up conditions are considered. Furthermore, Π should be especially important when scaling experiments are performed or different freeze-thaw processes are compared. In general, Π is thought to be the highest for small freezing distance containers such as freeze bags, due to short total freezing times. On the other end, passively cooled systems, such as freeze-thaw bottles, will have the lowest Π values due to their step-like cooling temperature decreasing and long freezing times. The authors therefore conclude, that from a cryo-concentration point of view, freezing temperature is highly important for systems with longer freezing times e.g., passively cooled systems and cooled vessels, which was also shown by Nidhi et al. (Miller et al., 2013). Contrarily, lowering the set temperature in fast freezing processes might only marginally improve bulk homogeneity and protein integrity as reported for freeze-thaw processes in single-use bags (Kolhe et al., 2012; Le Saout et al., 2012; Rayfield et al., 2017), with short freezing times, where transient starting conditions can play a significant role.

**TABLE 1:** Π values of the freezing device.

T_*set*_	Π /%
−60.0°C	46.6 ± 0.41
−50.0°C	31.8 ± 0.16
−40.0°C	19.2 ± 0.44
−30.0°C	8.8 ± 0.06
−25.0°C	6.4 ± 0.02
−22.5°C	6.0 ± 0.10
−20.0°C	5.4 ± 0.08

As an application example, the Plank model may be used for justification of a proven acceptable range in front of regulatory authorities, where lower freezing temperatures result in a wider acceptable range and thus a more robust process.

### Sample Temperature

The parallel, perpendicular freezing fronts during the freezing observed in our setup provide valuable information on the scalability of our process. It highlights the minimized boundary effects present in our setup, which is essential for small scale models. In small scale processes with uncontrolled boundary effects such as passive freezing in bottles, freezing from the bottom occurs, which may impact the freezing front shape and the final concentration profile as seen by Kolhe and Badkar (2011), where the bottom concentration was usually lower than the layer above. In studies using actively cooled systems, boundary effects are not yet described.

Another phenomenon observed was, that the distance of the LPTF was closer to the inner cooling wall, which is a result of the smaller heat transfer area at the inner wall. A simplified energy balance between the volume from the inner cooling wall to the LPTF and the outer cooling wall and the LPTF (see Supplementary Material) leads to a theoretical LPTF distance to the inner wall of 24 mm, which agrees with our findings.

Apparent descending of the LPTF to the bottom of a chamber when freezing at elevated temperatures can be explained by settlement of the freeze concentrated liquid throughout the process (Singh and Nema, 2010; Kolhe and Badkar, 2011). Concentrated solution pushed to the top at the LPTF agrees with results of Hauptmann et al. (2019). Freeze concentration leads to viscosity and density gradients in the solution inducing natural convection (Butler, 2002). Furthermore, freezing-point depression occurs with increased concentration. Thus, the LPTF position will sink to the bottom, when freeze concentration is superior at elevated freezing temperatures. This effect is likely to be more pronounced in large scale applications, due to an increased convection (Authelin et al., 2020) and larger sedimentation distances. A temperature probe is placed commonly at the LPTF for process monitoring (Lashmar et al., 2007; Le Saout et al., 2012), which might result in false results when different formulations are frozen. We therefore suggest the derivative based method discussed above for process characterization based on freezing time because of the method’s flexibility with regards to position of temperature probes in the container.

### Frozen Bulk Analysis

In general, our findings with concentration maxima of up to 2.5-fold agree with literature with actively cooled freezing devices (Webb et al., 2002; Rodrigues et al., 2011; Reinsch et al., 2015), who reported 1.3 to 2.5-fold freeze concentrations. The concentration gradient observed can be explained by the freeze-concentration due to solute exclusion (Roessl et al., 2014; Authelin et al., 2020) and the previously mentioned settlement of denser freeze concentrate (Kolhe and Badkar, 2011). The smaller reduction in bulk homogeneity when decreasing the temperature by 20°C at lower freezing temperatures can be attributed to smaller reduction in freezing time at lower temperatures due to physical limitations as seen by Plank’s model. This supports our findings, that freeze concentration and therefore freezing processes can be characterized by freezing time, which agrees with Hauptmann et al. (2019).

## Conclusion

The presented results give industrially relevant guidance for freeze-thaw process design and monitoring. A novel freeze-thaw device using two individual cooling circuits is demonstrated. While the model has an increased engineering complexity, it is capable of reducing boundary effects such as freezing from the bottom. Thus, process parameters, such as temperature dependent settlement of the LPTF, can be evaluated at a small scale, which is important for process monitoring. Furthermore, a high-resolution temperature monitoring approach with process interference was combined with a derivative-based method to calculate total freezing times. The determined freezing time had a high impact on the resulting freeze concentration profile of the frozen bulk. These freezing times can be estimated for a given actively cooled system using the Plank equation by model calibration with few freezing time experiments. However, for real processes at low freezing temperatures, the Plank model has to be extended by the non-dimensional number Π to consider start-up conditions present during cooldown of the heat transfer fluid and the system. Π might explain, why freezing temperature plays a more important role in short distance freezing processes. Thus, the reduction of freezing temperatures might have a bigger impact on frozen bulk homogeneity for freezing processes with larger characteristic distances as seen in stainless steel vessel. Processes with shorter freezing distances such as freeze bags might not be as improved by low freezing temperatures. These findings thus have a high impact on future process analytical technology strategies for freeze-thaw operations in the pharmaceutical industry.

## Data Availability Statement

The raw data supporting the conclusions of this article will be made available by the authors, without undue reservation.

## Author Contributions

JH initiated and supervised the work. DW evolved the concepts and set-up presented in this manuscript, performed the experimental work, analyzed and interpreted the data, and drafted the manuscript. Both authors read and approved the final manuscript.

## Conflict of Interest

The authors declare that the research was conducted in the absence of any commercial or financial relationships that could be construed as a potential conflict of interest.

## Abbreviations

LPTF: last point to freeze
PTFE: polytetrafluoroethylene
RMSE: root-mean-square error
TRIS: Tris-(hydroxymethyl)-aminomethane.

## References

[B1] Authelin J. R. Rodrigues M. A. Tchessalov S. Singh S. K. McCoy T. Wang S. (2020). Freezing of biologicals revisited: scale, stability, excipients, and degradation stresses. *J. Pharm. Sci*. 109 44–61. 10.1016/j.xphs.2019.10.062 31705870

[B2] Bauer K. C. Suhm S. Wöll A. K. Hubbuch J. (2017). Impact of additives on the formation of protein aggregates and viscosity in concentrated protein solutions. *Int. J. Pharm.* 516 82–90. 10.1016/j.ijpharm.2016.11.009 27836754

[B3] Bhatnagar B. S. Bogner R. H. Pikal M. J. (2007). Protein stability during freezing: separation of stresses and mechanisms of protein stabilization. *Pharm. Dev. Technol.* 12 505–523. 10.1080/10837450701481157 17963151

[B4] Butler M. F. (2002). Freeze concentration of solutes at the Ice/Solution interface studied by optical interferometry. *Cryst. Growth Des.* 2 541–548. 10.1021/cg025591e

[B5] Chang B. S. Kendrick B. S. Carpenter J. F. (1996). Surface-induced denaturation of proteins during freezing and its inhibition by surfactants. *J. Pharm. Sci.* 85 1325–1330. 10.1021/js960080y 8961147

[B6] Chang L. Shepherd D. Sun J. Ouellette D. Grant K. L. Tang X. C. (2005). Mechanism of protein stabilization by sugars during freeze-drying and storage: native structure preservation, specific interaction, and/or immobilization in a glassy matrix?. *J. Pharm. Sci.* 94 1427–1444. 10.1002/jps.20364 15920775

[B7] Fan T. H. Li J. Q. Minatovicz B. Soha E. Sun L. Patel S. (2018). Phase-field modeling of freeze concentration of protein solutions. *Polymers* 11 1–20. 10.3390/polym11010010 30959994 PMC6401895

[B8] Hauptmann A. Hoelzl G. Loerting T. (2019). Distribution of protein content and number of aggregates in monoclonal antibody formulation after large-scale freezing. *AAPS PharmSciTech* 20 1–11. 10.1208/s12249-018-1281-z 30631964 PMC6373418

[B9] Kolhe P. Badkar A. (2011). Protein and solute distribution in drug substance containers during frozen storage and post-thawing: a tool to understand and define freezing-thawing parameters in biotechnology process development. *Biotechnol. Prog.* 27 494–504. 10.1002/btpr.530 21302371

[B10] Kolhe P. Mehta A. Lary A. Chico S. Singh S. K. (2012). Large-scale freezing of biologics (Part III) understanding frozen-state protein and solute concentration changes in celsius bags. *BioPharm. Int.* 25 40–48.

[B11] Kumano H. Asaoka T. Saito A. Okawa S. (2007). Study on latent heat of fusion of ice in aqueous solutions. *Int. J. Refrig.* 30 267–273. 10.1016/j.ijrefrig.2006.07.008

[B12] Lashmar U. T. Vanderburgh M. Little S. J. (2007). Bulk Freeze–Thawing of Macromolecules. *BioProcess Int.* 5 44–54.

[B13] Le Saout X. Youssef E. Broly H. Costioli M. D. (2012). Safe Freeze-Thaw of Protein Drug Products?: a QbD Approach. *BioPharm. Int.* 23 28–43.

[B14] Maity H. Karkaria C. Davagnino J. (2009). Mapping of solution components, PH changes, protein stability and the elimination of protein precipitation during freeze-thawing of fibroblast growth factor 20. *Int. J. Pharm.* 378 122–135. 10.1016/j.ijpharm.2009.05.063 19505546

[B15] Martin H. (ed.) (2010). *Transient Conduction in Stagnant Media. VDI Heat Atlas. 2nd intern*. Berlin: Springer, 10.1007/978-3-540-77877-6

[B16] Miller M. A. Rodrigues M. A. Glass M. A. Singh S. K. Johnston K. P. Maynard J. A. (2013). Frozen-state storage stability of a monoclonal antibody: aggregation is impacted by freezing rate and solute distribution. *Int. J. Pharm. Sci.* 102 1194–1208. 10.1002/jps.23473 23400717

[B17] Pham Q. T. (2005). *Mathematical Modeling of Freezing Processes. Handbook of Frozen Food Processing and Packaging.* England, UK: Routledge, 141–174. 10.1201/b11204-9

[B18] Plank R. (1913). Die gefrierdauer von eisblocken. *Zeitschrift Für Die Gesamte Kälteindustrie* 20 109–114.

[B19] Privalov P. L. (1990). Cold denaturation of protein. *Crit. Rev. Biochem. Mol. Biol.* 25 281–306. 10.3109/10409239009090612 2225910

[B20] Randall M. Rossini F. D. (1929). Heat Capacities in Aqueous Salt Solutions. *J. Am. Chem. Soc.* 51 323–345. 10.1021/ja01377a001

[B21] Rayfield W. J. Kandula S. Khan H. Tugcu N. (2017). Impact of Freeze/Thaw Process on Drug Substance Storage of Therapeutics. *J. Pharm. Sci.* 106 1944–1951. 10.1016/j.xphs.2017.03.019 28343990

[B22] Reinsch H. Spadiut O. Heidingsfelder J. Herwig C. (2015). Examining the freezing process of an intermediate bulk containing an industrially relevant protein. *Enzyme Microb. Technol.* 71 13–19. 10.1016/j.enzmictec.2015.01.003 25765305 PMC4370381

[B23] Rodrigues M. A. Miller M. A. Glass M. A. Singh S. K. Johnston K. P. (2011). Effect of freezing rate and dendritic ice formation on concentration profiles of proteins frozen in cylindrical vessels. *J. Pharm. Sci.* 100 1316–1329. 10.1002/jps 24081467

[B24] Roessl U. Humi S. Leitgeb S. Nidetzky B. (2015). Design of experiments reveals critical parameters for pilot-scale freeze-and-thaw processing of L-lactic dehydrogenase. *Biotechnol. J.* 10 1390–1399. 10.1002/biot.201400766 25820483

[B25] Roessl U. Jajcevic D. Leitgeb S. Khinast J. G. Nidetzky B. (2014). Characterization of a laboratory-scale container for freezing protein solutions with detailed evaluation of a freezing process simulation. *J. Pharm. Sci.* 103 417–426. 10.1002/jps.23814 24338205

[B26] Singh S. K. Kolhe P. Wang W. Nema S. (2009). Large-Scale Freezing of Biologics. *BioProcess Int.* 7 32–44.

[B27] Singh S. K. Nema S. (2010). *Freezing and Thawing of Protein Solutions. Formulation and Process Development Strategies for Manufacturing Biopharmaceuticals.* Hoboken: Wiley, 625–675. 10.1002/9780470595886.ch26

[B28] Webb S. D. Webb J. N. Hughes T. G. Sesin D. F. Kincaid A. C. (2002). Freezing bulk-scale biopharmaceuticals using common techniques - and the magnitude of freeze-concentration. *BioPharm* 15 22–34.

[B29] Wöll A. K. Desombre M. Enghauser L. Hubbuch J. (2019a). A phase diagram-based toolbox to assess the impact of freeze/thaw ramps on the phase behavior of proteins. *Bioprocess Biosyst. Eng.* 43 179–192. 10.1007/s00449-019-02215-5 31563976

[B30] Wöll A. K. Schütz J. Zabel J. Hubbuch J. (2019b). Analysis of phase behavior and morphology during freeze-thaw applications of lysozyme. *Int. J. Pharm.* 555 153–164. 10.1016/j.ijpharm.2018.11.047 30458258

[B31] Zbacnik T. J. Holcomb R. E. Katayama D. S. Murphy B. M. Payne R. W. Coccaro R. C. (2017). Role of Buffers in Protein Formulations. *J. Pharm. Sci.* 106 713–733. 10.1016/j.xphs.2016.11.014 27894967

