# Supplementary material for: Temperature Based Process Characterization of Pharmaceutical Freeze-Thaw Operations
Source: Front Bioeng Biotechnol. 2021 Apr 9;9:617770. doi: 10.3389/fbioe.2021.617770 (PMC8062970; doi:10.3389/fbioe.2021.617770)
Supplement: Supplementary file 1 [file Data_Sheet_1.docx]

Supplementary Material

# Supplementary Data

LPTF calculation

An energy balance is used to calculate the theoretical LPTF distance to the inner wall d_LPTF, in._ Therefore, the sample volume is divided at the LPTF into an inner V_in_ and outer V_out_ volume with an equal freezing time t_freezing_.

| $t_{freezing,in}=t_{freezing,out}$ | I |
| --- | --- |

The heat flow $\dot{Q}$ into the sample can be calculated from the heat transfer coefficient k, the heat conducting surface A_Wall_ and the temperature difference between the sample and the cooling fluid $\Delta T_{\infty}$ as described by equation II.

| $\dot{Q}=k A_{wall} \Delta T_{\infty}$ | II |
| --- | --- |

The total enthalpy H that is removed within t_freezing_ is calculated by equation III,

| $\dot{Q} t{}_{freezing}=H=V \left( c_{p,ice} \Delta T_{sample,mean}+\rho_{l} \Delta h_{m} \right)$ | III |
| --- | --- |

where V is a sub volume, $\Delta T_{sample,mean}$ is the mean temperature difference between the mean sample temperature at equilibration and at t_freezing_, $\rho_{l}$ is the liquid sample density, $\Delta h_{m}$ is the specific latent heat. Equation IV can be calculated by solving I with II and III and calculating the specific areas and volumes with the inner r_in_ and outer r_out_ radii of the freezing device.

| $\frac{V_{in,LPTF}}{A_{in}}=\frac{V_{out,LPTF}}{A_{out}}$ | II & III in I |
| --- | --- |
| $r_{LPTF}= \sqrt{r_{in}r_{out}} with d_{LPTF}=r_{LPTF}-r_{in}$ | IV |

$\Delta T_{\mathrm{sample}}$ was assumed to be linear.
